# Determinants of Persistence and Tolerance of Carnivores on Namibian Ranches: Implications for Conservation on Southern African Private Lands

**DOI:** 10.1371/journal.pone.0052458

**Published:** 2013-01-09

**Authors:** Peter Andrew Lindsey, Carl Peter Havemann, Robin Lines, Lucille Palazy, Aaron Ernest Price, Tarryn Anne Retief, Tiemen Rhebergen, Cornelis Van der Waal

**Affiliations:** 1 Mammal Research Institute, Department of Zoology and Entomology, University of Pretoria, Pretoria, South Africa; 2 Panthera, New York, New York, United States of America; 3 Namibian Nature Foundation, Windhoek, Namibia; 4 Biométrie et Biologie Evolutive, Villeurbanne, France; 5 Nebraska Department of Environmental Quality, Lincoln, Nebraska, United States of America; 6 Department of Plant Production Systems, Wageningen University, Wageningen, The Netherlands; 7 Vanderwaal & Associates Agri-ecological Services, Omaruru, Namibia; Australian Wildlife Conservancy, Australia

## Abstract

Changing land use patterns in southern Africa have potential to dramatically alter the prospects for carnivore conservation. Understanding these influences is essential for conservation planning. We interviewed 250 ranchers in Namibia to assess human tolerance towards and the distribution of large carnivores. Cheetahs (*Acinonyx jubatus*), leopards (*Panthera pardus*) and brown hyaenas (*Hyaena brunnea*) were widely distributed on Namibian farmlands, spotted hyaenas (*Crocuta crocuta*) had a narrower distribution, and wild dogs (*Lycaon pictus*) and lions (*Panthera leo*) are largely limited to areas near source populations. Farmers were most tolerant of leopards and least tolerant of lions, wild dogs and spotted hyaenas. Several factors relating to land use correlated consistently with carnivore-presence and landowner tolerance. Carnivores were more commonly present and/or tolerated where; wildlife diversity and biomass were higher; income from wildlife was higher; income from livestock was lower; livestock biomass was lower; in conservancies; game fencing was absent; and financial losses from livestock depredation were lower. Efforts to create conditions whereby the costs associated with carnivores are lowest, and which confer financial value to them are likely to be the most effective means of promoting carnivore conservation. Such conditions are achieved where land owners pool land to create conservancies where livestock are replaced with wildlife (or where livestock husbandry is improved) and where wildlife generates a significant proportion of ranch income. Additional measures, such as promoting improved livestock husbandry and educational outreach efforts may also help achieve coexistence with carnivores. Our findings provide insights into conditions more conducive to the persistence of and tolerance towards large carnivores might be increased on private (and even communal) lands in Namibia, elsewhere in southern and East Africa and other parts of the world where carnivore conservation is being attempted on private lands.

## Introduction

Large carnivores are among the most challenging taxa to conserve. They are wide-ranging and are commonly persecuted [1]. For viable large carnivore populations to endure, they need extensive areas with sufficient prey and (usually) few people [2]. Human tolerance of carnivores can be affected by a variety of factors, including financial impacts imposed through losses of livestock or wildlife, or through misconceptions and prejudice. Historically, prejudice towards carnivores was reflected in state-sponsored persecution campaigns in some places [3]. Over 5,000 wild dogs *Lycaon pictus* and spotted hyaenas *Crocuta crocuta* were destroyed in Rhodesia during 1956 and 1961, for example [4], and in the Cape Province of South Africa, bounties were paid for the destruction of ∼343,000 black backed jackals *Canis mesomelas* and caracals *Caracal caracal* and 543 leopards *Panthera pardus* during 1914 and 1923 [5]. Similarly, in Namibia, 156 wild dogs were killed by wildlife management authorities during 1965–1966 [6].

Though state-sponsored carnivore control is largely a thing of the past, human-wildlife conflict and lethal control of carnivores is perhaps the primary threat facing carnivores in Africa [7]. Human-wildlife conflict often translates into lethal control, either pre-emptively, or in response to livestock or other losses, which can have severe impacts on carnivore populations [8]. Due to their tendency to range widely, even populations of carnivores occurring in protected areas are often subjected to lethal control [9]. Furthermore, the large majority of the distributions of all large African carnivores fall outside of protected areas [10]–[12]. Finding means to promote coexistence between carnivores and people is thus essential for their conservation, though that will be challenging, particularly for species such as lions.

The negative impact of humans on carnivores in much of Africa is probably increasing as human populations expand into previously wild areas, as natural habitat becomes fragmented and as prey populations decline [3]. In some parts of southern Africa, however, the development of transfrontier conservation areas, and wildlife-based land uses across large swathes of private and communal land have the potential to improve their conservation prospects [13], [14]. These developments have resulted in increasing wildlife populations outside of protected areas and incentives for landowners to conserve carnivores for ecotourism and trophy hunting in some instances [14]. In South Africa, Namibia and Zimbabwe for example, the devolution of user-rights over wildlife to landholders brought about the development of wildlife-ranching over vast areas (205,000, 287,000 and 27,000 km^2^ [prior to land ‘reform’] respectively), which has been associated with major increases in wildlife populations and shifts in species’ distributions [15]. These land use changes have created significant potential for the conservation of carnivores outside of protected areas. However, they have also created new conservation challenges, such as conflict with humans over valuable wildlife species [14].

In many areas successful carnivore conservation will be dependent on achieving coexistence between carnivores and people. Consequently, efforts to increase human tolerance for carnivores should be a central component of conservation efforts. A first step, however, is to understand existing attitudes towards carnivores and the reasons for them. A variety of studies have assessed human attitudes towards carnivores in Europe (e.g. [16]; [17]) and the USA [18]–[19]. In Africa, several authors have assessed the attitudes of commercial ranchers and communal farmers towards carnivores, though most of these studies have focused on individual species such as cheetahs *Acinonyx jubatus* [20], wild dogs [21] and lions *Panthera leo* [22], and/or on restricted study sites, including Kruger National Park in South Africa [23], North West province of South Africa [24], Laikipia District in Kenya [25], Serengeti National Park, [26], Ghanzi [27], Makgadigadi in Botswana [22] and on commercial conservancies in Namibia [28].

We assessed the distribution and status of six large carnivore species on Namibian commercial farmlands and identified correlates of persistence and landowner tolerance toward those species. These data are used to identify steps required to improve the conservation status of carnivores in Namibia and provide insights that are of relevance to conservation efforts elsewhere in southern and East Africa. Human-carnivore conflict is a globally significant issue [29], and our results are likely to be of interest anywhere that carnivore conservation on private lands is being attempted.

## Methods

### 1. Study Area

In Namibia, freehold farmland consists of ∼3,500 farms encompassing 353,533 km^2^ and comprising 43% of Namibia’s surface area in the central and southern part of the country [30]. Livestock production is the dominant land use on Namibian freehold farms, and is practised by 92.3% of landowners [15]. Wildlife-based land uses (which are often combined with livestock farming) are practised by ∼75% of land owners and occur on ∼287,000 km^2^ of freehold farmland. Exclusive wildlife based land use occurs on ∼32,000 km^2^ [15]. Twenty-five conservancies have been developed where multiple landowners manage wildlife cooperatively (comprising 1,008 farms and ∼43,250 km^2^) [15]. Conservancies in Namibia are defined as ‘a legally protected area of a group of *bona fide* land occupiers practicing cooperative management based on a sustainable utilization strategy, promoting conservation of natural resources and wildlife, and striving to reinstate the original biodiversity with the basic goal of sharing resources amongst all members’ (http//:www.canam.iway.na, accessed May 2010). The majority of farms (88.7%) have livestock-proof fencing, even in conservancies, though unlike the situation on ranch lands in South Africa, wildlife-proof fencing is relatively uncommon (occurring on 26.8% of farms; [15] [31]).

### 2. Data Collection

Approval for our study was obtained from the Namibian Ministry of Environment and Tourism. A structured, pre-tested questionnaire was used to gather data on land use, wildlife, presence/absence of carnivores, whether farmers wished to have carnivores on their property, and estimates of financial losses through depredation by carnivores on livestock. Data on presence/absence and tolerance were collected for cheetahs, brown hyaenas, spotted hyaenas, leopards, lions and wild dogs.

Sixty of the 104 commercial farmers unions in Namibia were randomly selected and farmers’ contacts requested (following [15]). From each union selected, four farmers were randomly sampled and contacted to request an in-person interview. Farmers were asked to provide verbal consent for the interview survey, and records were kept of negative responses to allow calculation of the refusal rate. Respondents were informed that all survey data would be kept anonymous, and ranchers’ names were not recorded. If respondents were not reachable, alternatives were randomly selected. Interviews were conducted in English, Afrikaans or Herero by four trained interviewers.

Two-hundred and fifty farmers were interviewed. Due to multiple farm ownership/lease-holding, the sample covered 412 farms (28,038 km^2^). There are believed to be 3,500 commercial management units in Namibia (Namibian Agricultural Union, pers. comm., 2010), so the sample comprised 11.8% of the ‘population’. Refusal rate was 4.8%, which is unlikely to introduce significant non-response bias [32]. In the initial sample, farmers in conservancies were over-represented, and such samples were randomly selected and removed (*n* = 82 removed) until the proportions reflected reality, to allow calculation of the percent occurrence of those species and the proportion of farmers tolerating them.

Wildlife and livestock biomass were estimated by multiplying the mean mass of individuals of a species (0.75 of standard female mass; [33] by respondents’ estimates of the populations of those species on their properties. The number of wild ungulate species was recorded and is referred to as ‘wildlife diversity’.

### 3. Statistical Analyses

We first tested for pair-wise correlations between our continuous variables using a Spearman non-parametric test to allow for the removal of redundant variables and ensure that each variable contributed unique insights. Survey data were then analysed using multiple logistic regressions [34]. When commencing with multiple logistic regressions, all variables expected to influence the dependent variable were included in the models and removed following a backwards stepwise procedure until all remaining variables were statistically significant. Rainfall data (which were obtained from a Namibian government database, http://www.met.gov.na/Pages/DefaultNew.aspx, accessed June 2011) were available in categorical form, and were reorganised into 4 categories (<200 ml/year, 201–300 ml/year, 301–400 ml, and >400 ml).

## Results

### 1. Relationships among Explanatory Variables

Several variables were significantly correlated with one another: the percentage of income from livestock was negatively correlated with income from wildlife (R^2^ = −0.64, *p*<0.001), wildlife biomass (R^2^ = −0.39, *p*<0.001), and wildlife diversity (R^2^ = −0.42, *p*<0.001) and positively correlated with livestock biomass (R^2^ = −0.37, *p*<0.001); income from wildlife was positively correlated with wildlife biomass (R^2^ = 0.54, *p*<0.001) and diversity (R^2^ = 0.66, *p*<0.001); and, wildlife biomass and diversity were positively correlated (R^2^ = 0.71, *p*<0.001). Consequently, we selected one of the correlated variables as a proxy for the others in order to avoid redundant information in our subsequent analysis. For the models used to assess predictors of large carnivore presence/absence, wildlife biomass was used as the proxy for income from livestock, livestock biomass, income from wildlife and wildlife diversity, which were all excluded from the models. Wildlife biomass was selected as it was considered to be the most biologically relevant factor and the one most likely to influence carnivore distribution. For the model used to assess predictors of tolerance, income from wildlife was used as the proxy as that was considered likely to be the most direct determinant of tolerance: our hypothesis was that those receiving income from wildlife would be more tolerant towards carnivores, as was found by [31].

### 2. Presence/absence of Carnivores

**Figure 1:**
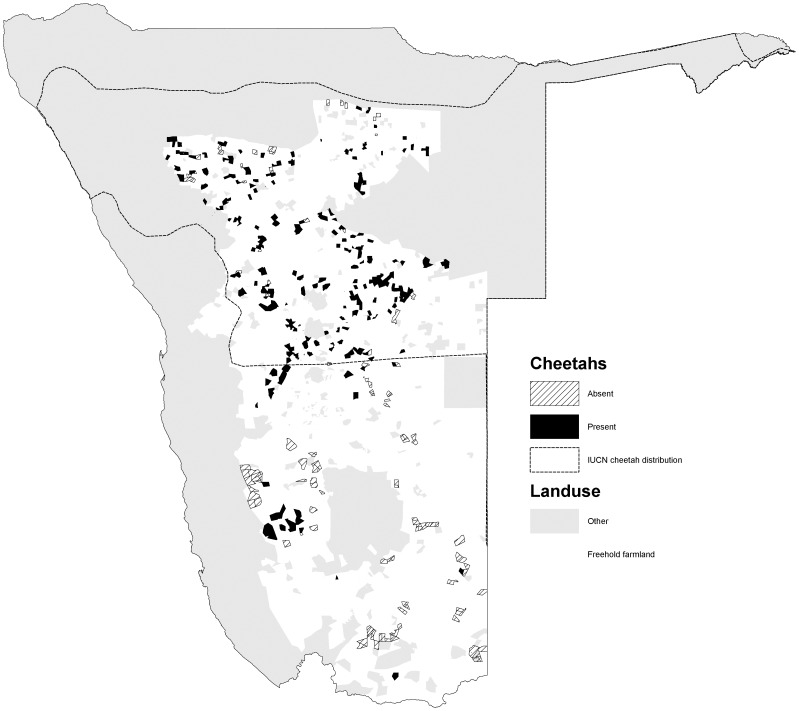
Distribution of cheetahs on commercial farmlands in Namibia.

**Figure 2:**
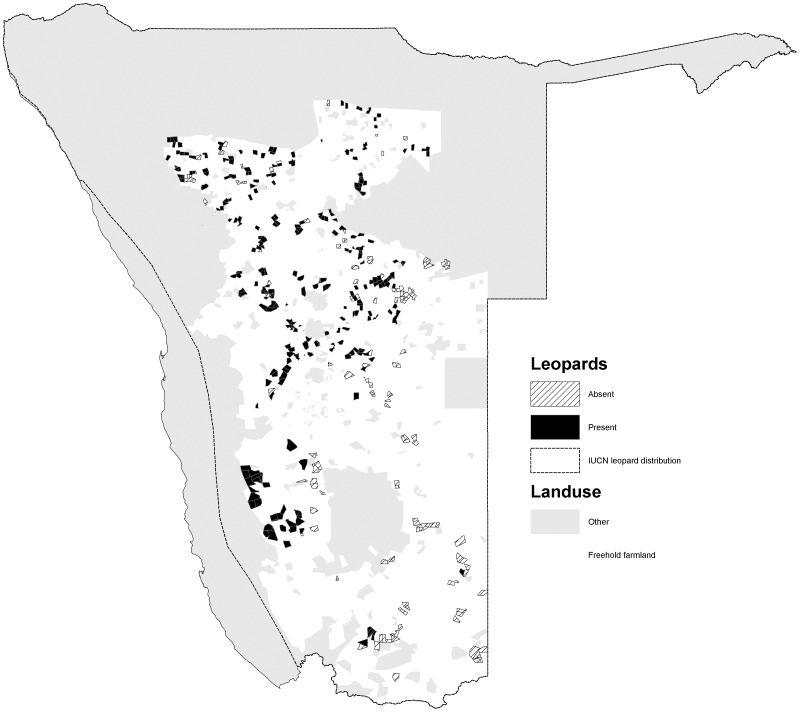
Distribution of leopards on commercial farms in Namibia.

**Figure 3:**
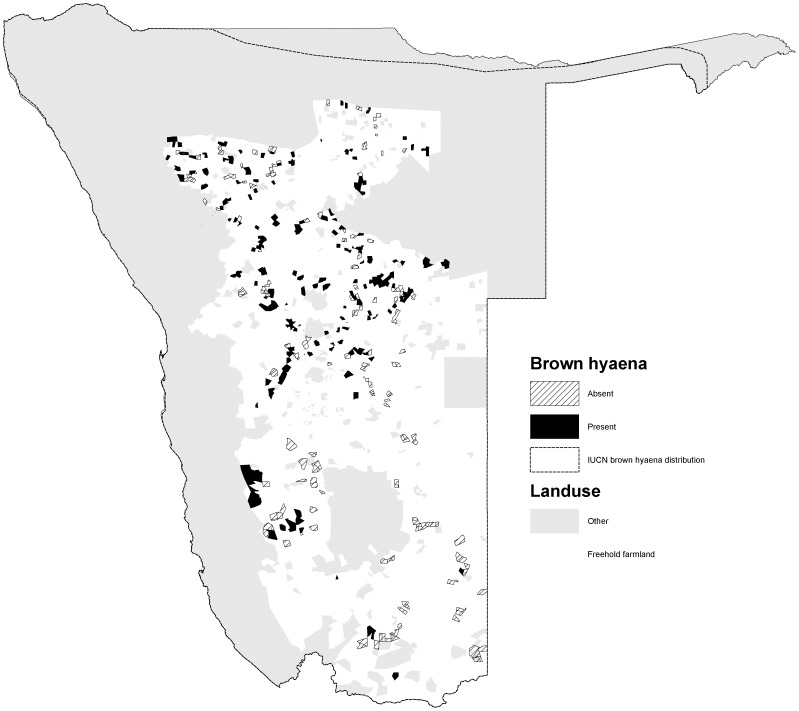
Distribution of brown hyaenas on Namibian commercial farms.

**Figure 4:**
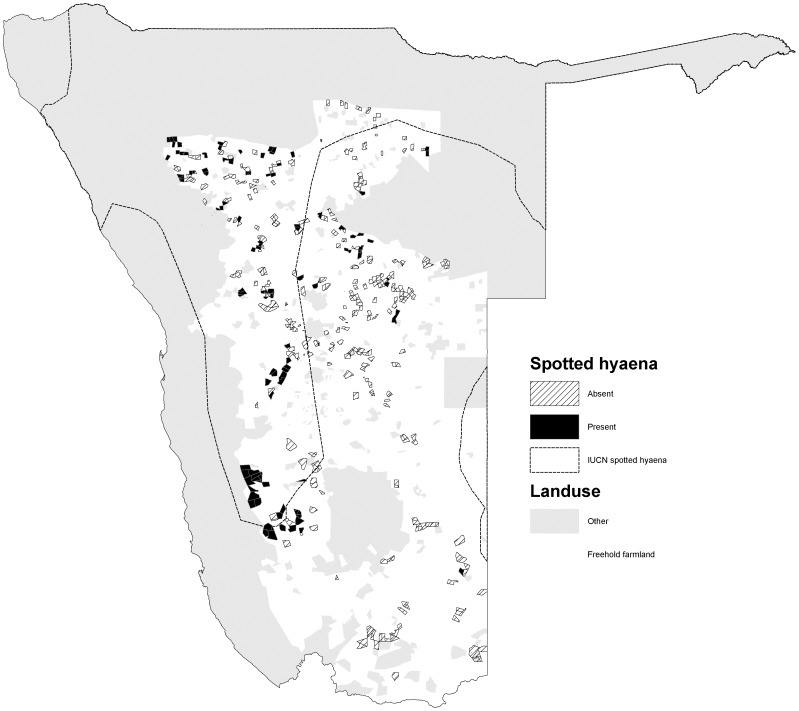
Distribution of spotted hyaenas on commercial farms in Namibia.

**Figure 5:**
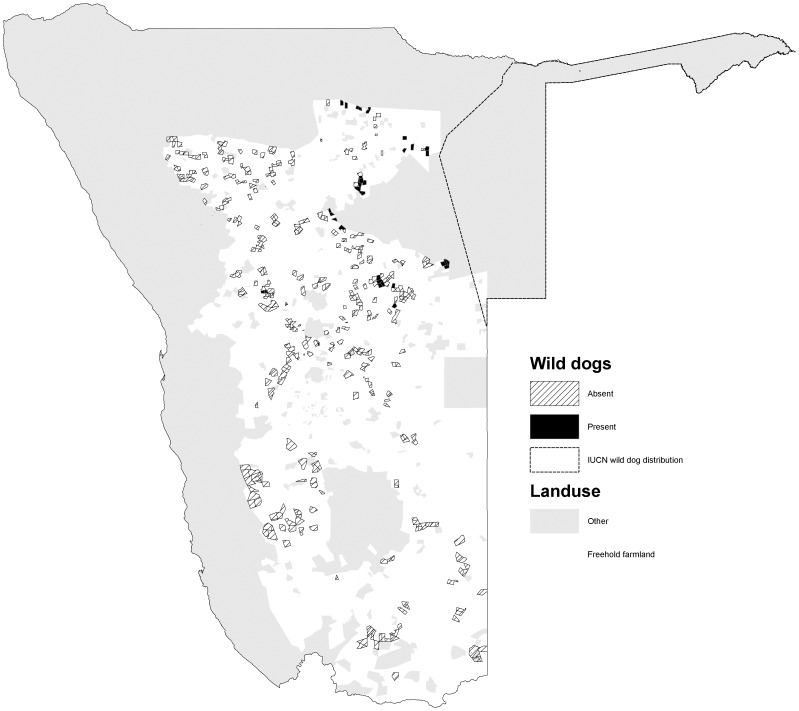
Distribution of wild dogs on Namibian commercial farms.

**Figure 6:**
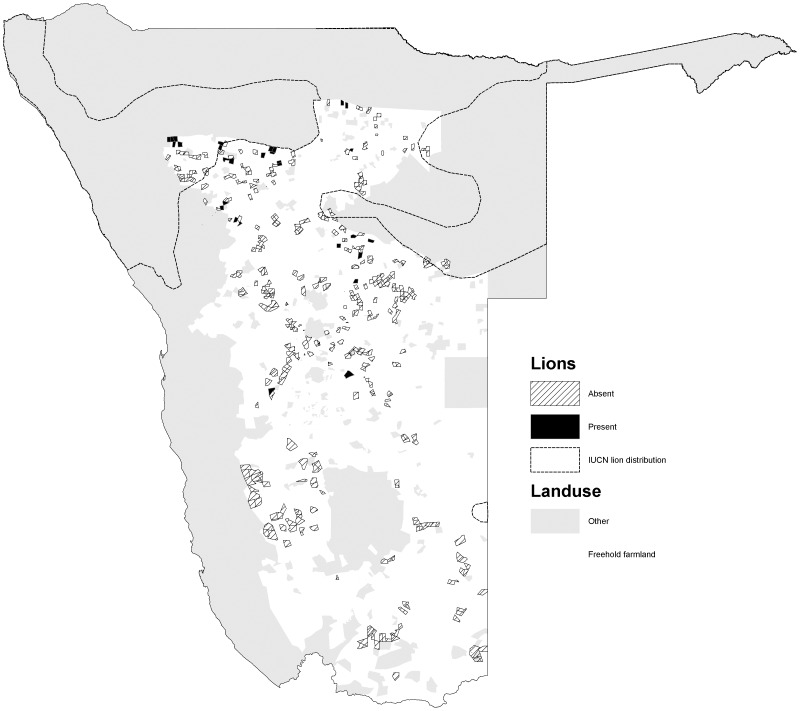
Distribution of lions on commercial farms in Namibia.

The percentage occurrence of carnivores varied among species (*χ2* = 586, *d.f.* = 5, *p<*0.001). Cheetahs had the widest occurrence (72.0% of farms) and were widely distributed in the northern, central and south western parts of the commercial farming area (Figure 1). Leopards were also widely distributed (70.3%), with a similar distribution to cheetahs, but also occurred in the far south of the country (Figure 2). Brown hyaenas had a similar distribution to leopards, though with a lower frequency of occurrence (54.5%) (Figure 3). Spotted hyaenas were widely distributed in the northern, central and south western parts of the commercial farming area, but persisted on only a minority of farms (28.5%) (Figure 4). Wild dogs (9.7%) and lions (9.1%) had low percent occurrences and were restricted to the extreme north east and north respectively (with the exception of some isolated lion populations in the central part of the farming area) (Figures 5, 6).

The most commonly recorded combination of carnivore presence was cheetahs, brown hyaenas and leopards (Table 1). Wild dogs only occurred where at least three other carnivore species occurred, and lions only occurred where four other species occurred (Table 1).

**Table 1:** Combinations of the presence/absence of and tolerance toward large carnivores on Namibian farms.

Combination of carnivore species	% of farms on which combination occurs	% of farmers desiring the combination
Cheetahs/Brown hyaenas/Leopards	30.3	10.4
No large carnivores present	13.7	28.4
Cheetahs/Brown hyaenas/Spotted hyaenas/Leopards	12.4	0
Cheetahs/Leopards	7.9	4.1
Cheetahs/Spotted hyaenas/Leopards	6.2	3.1
Cheetahs	4.6	–
Cheetahs/Brown hyaenas/Spotted hyaenas/Leopards/Lions	4.1	–
Cheetahs/Brown hyaenas	4.1	–
Cheetahs/Brown hyaenas/Leopards/Wild dogs	2.9	–
Leopards	2.1	3.6
Brown hyaenas	–	9.9
Brown hyaenas/leopards	–	6.3
All large carnivores	–	5.8
Spotted hyaenas/Leopards	–	2.7
Cheetahs/spotted hyaenas/leopards/lions/wild dogs	–	2.7
Cheetahs/Brown hyaenas/Leopards/Spotted hyaenas	–	2.7
Others (in <5 farms)	11.7	20.3

There were a number of consistent patterns relating to the presence/absence of carnivores on farmlands. Carnivores were more commonly present where: wildlife biomass was higher (and thus where wildlife diversity and income from wildlife was higher, and income from livestock was lower); on farms in conservancies (except for wild dogs and lions); on farms where the species in question was wanted by the landowner; where farms were not surrounded by game fencing (except for brown hyaenas and wild dogs); on farms closer to protected areas (except for wild dogs); and, on smaller farms (except for brown hyaenas, spotted hyaenas and wild dogs) (Table 2). However, while the directions of these patterns were consistent across most species, not all of them were statistically significant for all species (Table 2). Leopard distribution was also affected by vegetation and the species was most commonly recorded on farms in Northern Kalahari vegetation (100% of farms), Western Highlands vegetation (94.4%) and Highland Shrublands (92.3%) and least commonly recorded in Karas Dwarf Shrublands (15.4%) and Dwarf Shrub (16.7%). The distribution of brown hyaenas was related to rainfall, with the species occurring more frequently in areas with 301–400 ml rain/year (77.8% of farms) and >400 ml/year (70.3%) than in areas with less rain (200–300 ml/year –62.3%; <200 ml/year –24.5%).

**Table 2:** Correlates of the presence/absence of large carnivores on Namibian farmlands (means ± SD) (bold/underlined values are those that were statistically significant (p≤0.05) following a multiple logistic regression.

	Cheetah		Leopard		B. hyaena		S. hyaena		Wild dogs		Lions	
	Present	Absent	Present	Absent	Present	Absent	Present	Absent	Present	Absent	Present	Absent
Overall	72.0	28.0	70.3	29.7	54.5	45.5	28.5	71.5	9.7	90.3	9.1	90.9
Indicators varying with species presence/absence												
Size of farm (km^2^)	**92.1±71.6**	**157±161**	99.1±96.2	123±107	115±95	98.9±102	131±213	105±104	108±102	72.2±55.0	**66.9±35.1**	**116±149**
Wildlife biomass (kg/km^2^)	1,407±1808	917±3,268	1,570±2,451	532±622	1,449±2,071	1,074±2,343	1,490±1,901	1,233±2,283	2,272±4,080	1,223±1,938	1,124±330	1,321±2,238
Distance to nearest park (km)	63.2±42.9	78.0±44.4	**55.6±36.5**	**95.7±47.8**	62.7±43.2	72.1±43.5	**48.1±32.3**	**72.7±45.1**	68.6±48.1	66.2±43.7	51.7±41.6	67.6±51.6
Rainfall a	Not significant	Not significant	Not significant	Not significant	**Significant**	**Significant**	Not significant	Not significant	Not significant	Not significant	Not significant	Not significant
Vegetation b	Not significant	Not significant	**Significant**	**Significant**	Not significant	Not significant	Not significant	Not significant	Not significant	Not significant	Not significant	Not significant
Species present/absent in:												
Farms in conservancies	**93.9%**	**6.1%**	**87.1%**	**12.9%**	**77.6%**	**22.4%**	28.5**%**	71. 5**%**	5.2**%**	94.8**%**	8.1%	91.9%
Farms not in conservancies	**67.2%**	**32.8%**	**63.4%**	**36.6%**	**46.6%**	**53.4%**	27.5**%**	72.5**%**	9.9**%**	90.1**%**	8.2%	91.8%
Where species is wanted	88.7%	11.3%	**84.5%**	**15.5%**	**72.7%**	**27.3%**	42.7**%**	57.3**%**	10.0**%**	90.0**%**	15.2%	84.8%
Where species is not wanted	74.3%	25.7%	**54.3%**	**45.7%**	**31.4%**	**68.6%**	22.5**%**	87.5**%**	6.0**%**	94.0**%**	7.5%	92.5%
Game fencing present	68.4**%**	31.6**%**	63.8**%**	36.2**%**	64.3**%**	35.7**%**	22.4**%**	87.6**%**	5.6**%**	94.4**%**	**4.3%**	**85.7%**
Game fencing absent	89.0**%**	11.0**%**	79.0**%**	21.0**%**	52.9**%**	47.1**%**	31.7**%**	68.3**%**	2.9**%**	97.1**%**	**11.5%**	**88.5%**
Statistical test results	*χ2* = 45.1, *d.f.* = 2, *p<*0.001		*χ2* = 84.7, *d.f.* = 13, *p<*0.001		*χ2* = 70.2, *d.f.* = 5, *p<*0.001		*χ2* = 13.5, *d.f.* = 1, *p<*0.001		No significant fit		*χ2* = 10.3, *d.f.* = 2, *p = *0.006	

a Categorized as: <200 ml/year; 200–300 ml/year; 301–400 ml/year; >400 ml/year.

b Comprising 11 vegetation categories.

### 3. Tolerance of Carnivores

**Figure 7:**
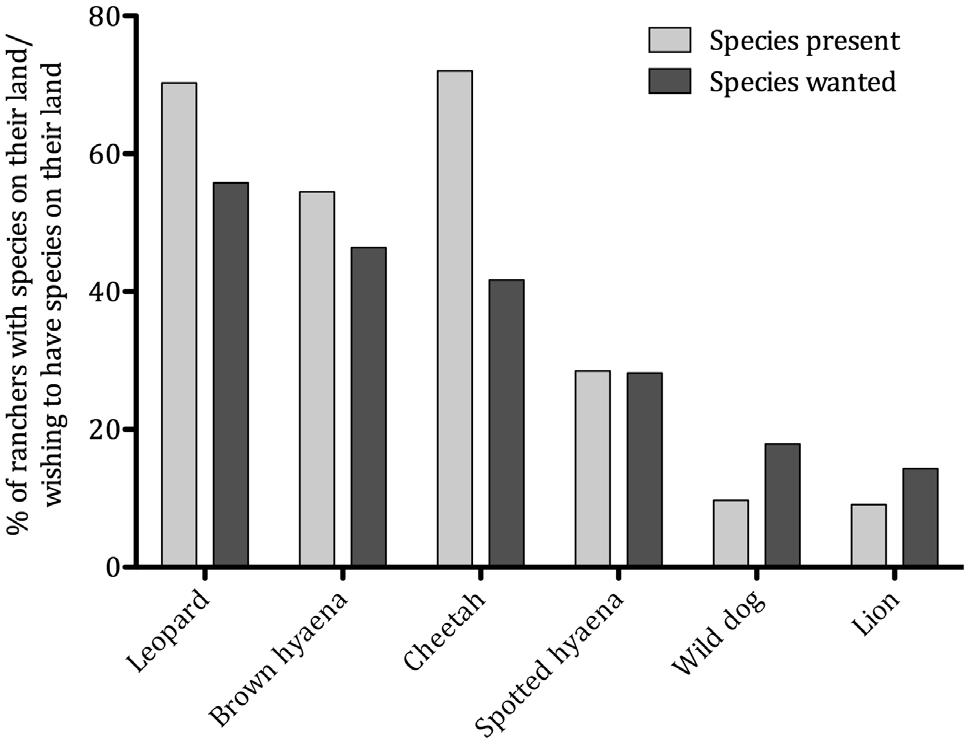
Percentage occurrence of large carnivore species on Namibian commerical farms and the percentage of farmers who wish to have those species on their properties.

Landowner tolerance towards carnivores varied with species (*χ^2^* = 178, *d.f.* = 7, *p<*0.001). Farmers were most tolerant of leopards, and least tolerant of lions, wild dogs and spotted hyaenas (Figure 7). The most common combination of carnivores that ranchers wanted to have on their land was brown hyaenas, cheetahs and leopards (Table 1). Lions and wild dogs were only desired by farmers who also wanted four other large carnivore species, suggesting that specific circumstances are required for tolerance towards those species or that only the most tolerant farmers are willing to accept those species (Table 2).

Carnivores were more commonly wanted where: income from wildlife was higher (and thus wildlife biomass and diversity were higher, and income from livestock was lower); farmers were younger, on the land of German/English speaking farmers (except for lions), where farmer’s landholdings were smaller; in conservancies; where game fencing was absent; and where financial losses due to depredation on livestock by the species in question was lower (except for lions) (Table 3).

**Table 3:** Correlates of whether Namibian farmers wished to have various species of carnivores on their land (means ± SD) (bold/underlined values are those that were statistically significant (p≤0.05) following a multiple logistic regression.

	Cheetah		Leopard		B. hyaena		S. hyaena		Wild dogs		Lions	
	Want	Don’t want	Want	Don’t want	Want	Don’t want	Want	Don’t want	Want	Don’t want	Want	Don’t want
% of farmers who want species	41.7	58.3	58.8	44.2	46.4	53.6	28.2	71.8	17.8	81.2	14.3	85.7
Indicators varying with species presence/absence												
Rancher age	**49.6±12.9**	**54.1±12.6**	50.6±12.9	54.2±50.6	50.8±12.8	54.1±12.8	**48.5±10.9**	**53.5±13.2**	**47.6±10.4**	**52.9±13.1**	49.4±11.6	52.6±13.0
Language – Black African	**38.7**	**61.3**	25.8	74.2	41.9	58.1	27.6	72.4	16.7	83.3	6.7	93.3
Language – Afrikaans	**31.6**	**68.4**	50.0	50.0	50.0	50.0	25.2	74.8	15.0	85.0	17.7	82.3
Language – German/English	**54.3**	**45.7**	73.1	26.9	71.3	28.7	32.2	67.8	20.9	79.1	12.9	87.1
Size of farm (km^2^)	100±85	110±112	105±86	110±112	**92.9±67**	**100±134**	108±87	114±113	87.4±59.66	112±109	106±88	108±104
% income from wildlife	**29.5±30.6**	**25.6±31.0**	**35.7±33.6**	**16.2±22.6**	**36.1±34.1**	**15.1±21.1**	**39.6±36.5**	**22.2±27.2**	**41.4±35.5**	**23.4±28.5**	**45.7±33.8**	**23.6±29.3**
Distance from nearest park (km)	**56.9±36.1**	**73.3±46.3**	60.7±39.0	73.5±47.1	64.8±42.0	069.5±45.7	62.1±38.3	69.1±45.9	52.8±33.2	69.5±44.9	59.8±33.8	67.5±44.8
Farms where species is wanted												
Where species is present	42.2%	57.8%	61.8%	38.2%	**76.1%**	**23.9%**	**27.6%**	**72.4%**	26.3%	73.7%	22.7%	77.3%
Where species is absent	38.3%	61.7%	40.7%	59.3%	**25.9%**	**74.1%**	**28.9%**	**71.1%**	17.4%	82.6%	13.7%	86.3%
Farms in conservancies	51.3%	48.7%	**71.8%**	**28.2%**	72.6%	27.4%	31.7%	68.3%	21.5%	78.5%	17.3%	82.7%
Farms not in conservancies	33.3%	66.7%	**57.4%**	**42.6%**	43.7%	56.3%	25.4%	74.6%	14.0%	86.0%	13.3%	86.7%
Game fencing present	39.1%	60.9%	44.1%	55.9%	20.2%	79.8%	25.2%	79.8%	14.8%	86.2%	13.0%	87.0%
Game fencing absent	43.8%	56.2%	65.9%	34.1%	50.8%	49.2%	35.6%	64.4%	20.8%	80.2%	15.4%	84.6%
Livestock losses (USD)	**2097±4669**	**4371±7641**	1871±2678	3675±9506	304±421	618±302	747±1622	2354±4040	0	0	7976±1347*	2708±3514*
Statistical test results	*χ2* = 31.8, *d.f.* = 5, *p<*0.001		*χ2* = 34.7, *d.f.* = 1, *p<*0.001		*χ2* = 67.7, *d.f.* = 3, *p<*0.001		*χ2* = 22.2, *d.f.* = 2, *p<*0.001		*χ2* = 14.8, *d.f.* = 2, *p = *0.016		*χ2* = 11.8, *d.f.* = 1, *p = *0.007	

• Based on low sample sizes (n = 10 respondents provided estimates of livestock losses to lions).

The most common reasons given by farmers for not wanting carnivores on their land were: that they kill livestock; they kill wildlife; and they impose financial costs (Table 4). Common reasons for wishing to have carnivores were: that they are not problematic or do not kill too much; due to their value for ecotourism; and (in the case of species other than wild dogs and brown hyaenas), their value for trophy hunting; and, due to their ecological role (Table 4). Some farmers were positive towards hyaenas because of their role in clearing the bush of carcasses as scavengers (Table 4).

**Table 4:** Explanations given by landowners when asked if they would like to have each species of large carnivore on their land.

Reasons given for attitudes	Wild dogs	Cheetahs	Leopards	Lions	Spotted hyaenas	Brown hyaenas
**Reasons for negative attitudes**						
They kill livestock	73.7	60.6	77.2	69.7	66.7	72.3
They kill a lot of/too much game	9.7	12.7	7.9	10.6	11.1	9.2
They kill small stock	9.1	8.5	22.8	7.6	5.6	7.7
They impose financial losses	15.1	11.3	7.9	10.6	18.9	13.4
They pose a risk to human safety	0.5	0	2	4.6	1.1	1.5
Damage fences	0	4.2	0	0	1.1	4.6
They kill every day	0	7.5	0	0	0	0
**Reasons for positive attitudes**						
They are no problem/do not kill too much	23.7	20.1	40.1	6.9	6.4	23.3
Their value for ecotourism	18.4	14.5	8.2	27.6	3.2	13.3
Their value for trophy hunting	0	8.1	10.7	6.9	9.7	1.7
Their ecological role/part of the system	29	12.9	19.3	20.7	12.9	15
Because they don’t waste	0	0	8.2	0	0	0
They clean the bush of carcasses	0	0	0	6.9	12.9	31.7
To assist their conservation	10.5	8.1	0.8	6.9	0	1.7
Control rodents and small animals	0	1.6	0	0	0	0
I like them	0	4.8	4.9	3.5	3.2	5
They are beautiful		6.5	0	10.3	3.2	0
Will want them if I go in for pure game farming	0	0	0.8	6.9	3.2	0
They use big areas	7.8	3.2	0	0	0	0

Lions, followed by cheetahs and leopards caused the largest estimated financial impacts due to livestock depredation whereas the estimated losses caused brown hyaenas were lower (Table 3). Financial impacts from livestock depredation were most commonly recorded for: lions (100% of farms where they occurred); black backed jackals (87.5% of farms); cheetahs (86.8%); leopards (82.1%); caracals (76.9%); spotted hyaenas (73.7%); and brown hyaenas (37.5%). No incidences of financial losses to wild dogs were reported by respondents.

## Discussion

### 1. Carnivore Distribution

The quality of our depictions of carnivore distribution is dependent on ranchers’ ability to determine whether each species is present/absent on their land. Ranchers are probably more likely to be unaware of species occurring at low densities or occasionally on their property than to wrongly believe that a species was present, and so if erroneous, our depictions of distribution are likely over-conservative. However, our depictions are consistent with those from other sources and likely provide a reasonable representation of the distribution of carnivores on Namibian ranches [35]–[37]. Future studies could improve on the reliability of our methods by testing the ability of ranchers to identify carnivores and discarding data from farmers who failed the test [25].

Among large carnivores, leopards, cheetahs and brown hyaenas have the widest distributions on Namibian freehold farmlands, sharing a similar pattern of occurrence. Large carnivores are largely absent from the southern farmlands due to the high prevalence of small-stock farming (likely to create conditions of intense conflict with farmers) and jackal-proof fencing present [36]. Namibia has the largest population of cheetahs in the world, most of which occur on farmlands [20]. Our findings reiterate the importance of Namibian farmlands for cheetah conservation, particularly given that protected areas encompass <5% of the population [36]. Namibian farmlands provide potentially ideal conditions for cheetahs due to high prey-densities and the scarcity of lions and spotted hyaenas, which affect cheetahs adversely through predation and kleptoparasitism [38]. Cheetah numbers in South Africa are believed to have increased in response to increasing wildlife populations due to the spread of wildlife ranching [37], [39], and the same may be occurring in Namibia [36]). The cheetah distribution extends further south than depicted by IUCN (2008) and includes areas that were previously identified as ‘possible’ and ‘recoverable’ range [11] (http://www.cites.org/cms/public/common/resources/annual_reports.pdf, accessed April 2012).

Our depiction of leopard distribution is similar to that of [35], though our records extend further east in the central-southern part of the commercial farming area. Our data suggest that leopards have a more restricted and patchy distribution than depicted by IUCN (2008), being generally absent from south eastern areas. Leopard densities on farmlands are higher than in parks in some areas, due to high prey abundance [40].

Brown hyaenas are thought to number between 500–1000 individuals in Namibia and are distributed ‘sporadically throughout the country’ [41]. Our data suggest that, in the central and northern sections of the commercial farming area, brown hyaenas are widespread, but have a more restricted distribution than depicted by IUCN (2008), being largely absent from the south east.

Spotted hyaenas also have a relatively wide distribution on Namibian farmlands, though with a lower percentage occurrence than smaller carnivores. Approximately 2,000–3,000 spotted hyaenas occur in Namibia, primarily in Etosha and Namib Naukluft national parks, the northern communal lands, and the eastern border with Botswana [41]. Our data suggest that spotted hyaenas are more widely distributed in the northern central farmlands than depicted by [41] or IUCN (2008) though in a similar pattern to that presented by [35].

Wild dogs and lions occur on <10% of Namibian farms and are restricted to the north eastern and northern areas, respectively. The wild dog distribution reflects the areas described as ‘resident’ and ‘possible’ by [11]. Lions were recorded more widely than reported by [35] or IUCN (2008), presumably as a result of sporadic reintroductions and spill over from Etosha National Park. Wild dogs are distributed slightly more widely than suggested by IUCN (2008), though sightings on commercial farmlands may be of dispersing groups rather than resident packs. We cannot say whether the differences in distribution for carnivores depicted by our study versus others are due to changes in populations or artefacts of different methodologies. However, the more fragmented nature of brown hyaenas and leopards suggests that their status may be more slightly more vulnerable than previously recognized, the status of lions and wild dogs similarly tenuous, and the conservation status of cheetahs and spotted hyaenas slightly healthier.

### 2. Correlates of Carnivore Occurrence

The presence of carnivores on Namibian farmlands was positively related to the development of wildlife-based land uses. Most species tended to be present where the wildlife biomass was higher, (and thus where wildlife diversity was higher, income from wildlife [via trophy hunting and to a lesser extent ecotourism and other forms of wildlife use] was higher and income from livestock was lower), where landowners have formed conservancies, and where game fencing was absent. Carnivores were more commonly present on farms where they are wanted by landowners, highlighting the importance of rancher attitudes in determining the fate of carnivores. Some carnivores were more commonly present on smaller farms, potentially because on smaller properties ranchers were more aware of what species occur on their land.

The distribution of wild dogs and lions (and to a lesser extent, spotted hyaenas), appear to be particularly influenced by human intolerance. Wild dogs occur at lower densities on farmlands (0.01–0.05 individuals/100 km^2^) than in the communal lands to the north east (1.0–1.4 individuals/100 km^2^), despite the comparatively lower prey densities in the latter (http://www.nnf.org.na/NNF_pages/wilddogproject.htm). Remaining populations of wild dogs and lions in Namibia are small (300–600, and 315–695, respectively) and restricted to areas near source populations, which include Etosha and Kaudom national parks, Kunene and Caprivi for lions, and the far north east, including Kaudom, Tsumkwe and Caprivi for wild dogs ( [11]; http://www.nnf.org.na/NNF_pages/wilddogproject.htm [10]).

### 3. Tolerance of Carnivores

Human tolerance is a key determinant of the conservation status of carnivores [42] and there is evidence of widespread lethal-control of carnivores on Namibian commercial farmlands. For example, 15% of farmers shoot leopards on sight and 60% shoot the species following livestock depredation [21]. Similarly, killing by humans is the main source of mortality for adult cheetahs on Namibian farmlands [43]. Approximately 120 cheetahs were removed per year during 1998–2000 [20]. At least 30 incidents of persecution of wild dogs were recorded over six years (R. Lines unpublished data). Similarly, at least 29 lions per year are killed on the commercial farms adjacent to Etosha National Park [35]. Furthermore, much anthropogenic mortality of carnivores likely goes unrecorded. For example, one rancher we interviewed claimed to have shot >200 cheetahs during his life.

Leopards and brown hyaenas are the most tolerated species among Namibian ranchers, in keeping with South Africa and Zimbabwe where leopards are the most accepted large carnivore [31]. Leopards are perceived to have value through ecotourism and trophy hunting, whereas brown hyaenas are perceived to impose few costs. Spotted hyaenas, wild dogs and lions were the least tolerated species, in keeping with South Africa and Zimbabwe (where lions and wild dogs were least popular) and Kenya (where spotted hyaenas were least popular) [25]. The low popularity of wild dogs was despite none of the farmers interviewed having recorded livestock losses to the species, suggesting that negative attitudes are related more to potential/perceived than actual impacts [44]. Lions, by contrast, were reported to cause severe financial impacts.

### 4. Possible Steps to Improve the Prospects of Carnivore Conservation

#### 4.1 Promoting conducive land uses

Tolerance towards carnivores was higher where income from wildlife is higher (and thus where wildlife diversity and biomass was higher, and where income from livestock was lower), in keeping with [31], [45] [27]. Promoting the development of wildlife-based land uses is thus likely to improve the prospects for carnivore conservation. The development of wildlife-ranching results in the recovery of prey populations. There are 1.8–2.8 million wild ungulates on Namibian farms and populations are increasing [15] in contrast to the declines observed in many other parts of Africa [46]. Increased populations of wild prey reduce the frequency of livestock depredation, thus improving the prospects for coexistence between people and carnivores ( [47]–[48]). With the development of wildlife-based land uses, wildlife including carnivores becomes financially valuable for ecotourism and/or trophy hunting under certain conditions [14]. There is a high willingness to pay among tourists to view carnivores, such as trips to see wild dogs at dens or leopards at bait sites [21], [49]. If landowners were to market carnivore viewing tours, potential earnings could help off-set losses through livestock depredation and improve tolerance [21].

Large carnivores are also important for trophy hunting ([50]–[51]). Cheetahs, leopards, spotted hyaenas, brown hyaenas and lions are hunted as trophies in Namibia. However, the value of cheetahs for trophy hunting is undermined by restrictions on the import of trophies into the US (the main market for trophy hunters) [52], and the value of lions is reduced by refusal of the Namibian government to issue trophy permits on farms near Etosha National Park. Rectifying both situations with due care could help create incentives for conservation of those species. In some scenarios however, trophy hunting can create conflict due to landowners objecting to the loss of potential hunting trophies to predators, particularly on small, fenced ranches (as explained below) [14].

#### 4.2 Promoting development of conservancies and limiting game fencing

Several conservancies have been formed in Namibia and in such areas carnivores are more frequently present and tolerated by land owners, in keeping with South Africa and Zimbabwe [31], [45]. Namibian conservancies typically retain both internal fencing and livestock, unlike those typical of Zimbabwe and South Africa [15]. Nonetheless, conservancies do confer increased tolerance to carnivores, possibly because of better livestock husbandry, reduced prevalence of game fencing, and increased importance of wildlife-based income [28]. Measures to promote the formation of fully-integrated conservancies where fencing and livestock is removed and ecotourism or low off-take trophy hunting developed would likely further benefit carnivores, and may encourage reintroductions of lions and wild dogs. Such conditions have arisen in many instances in South Africa and Zimbabwe, and large carnivores have been reintroduced widely [53]–[56] [57]–[58].

Where ranches are surrounded by game fencing, ranchers are less tolerant of carnivores in keeping with South Africa and Zimbabwe [31]. Game fencing is relatively rare in Namibia (as it is not a legal perquisite for the commercial utilization of wildlife). By contrast, in South Africa and western Botswana large areas of ranch land are fragmented with fencing, creating conditions where carnivores are commonly persecuted [14] [59]. Game fencing appears correlated with the utilization of high proportions of the sustainable yield of wild ungulates through hunting and in some instances, the reintroduction of particularly valuable, or predation-vulnerable exotic species, creating conditions conducive to conflict with carnivores [14] [60]. The Namibian government should discourage the development of wildlife-proof fencing around individual ranches.

#### 4.3 Livestock husbandry

Tolerance of carnivores was unsurprisingly higher where livestock losses were lower, and multiple studies indicate that reduced livestock depredation results in reduced human persecution of carnivores [20], [47], [48]. Financial losses due to livestock depredation reported by farmers in this study (US$2,644/farm to leopards) were higher than those reported (US$1,370/farm to leopards) [21]. Measures to reduce livestock depredation are essential for promoting carnivore conservation, and options include the use of herders, guarding dogs/donkeys, more effective corrals, synchronized calving, use of calving camps, mixing heifers with older and more experienced cows and leaving horns on some cows [21], [60]. Preventing financial losses on wildlife ranches is harder. Valuable prey species can be kept in carnivore-proof fenced camps, or forms of wildlife ranching such as ecotourism which confer value to carnivores can be encouraged.

#### 4.4. Other interventions

In some cases, persecution of carnivores arises from misconceptions regarding the impacts of carnivores, and prejudice towards a particular species [44]. Education and outreach efforts involving landholders are thus important [20]. A potential additional strategy would be to reward landowners (or conservancies) for the successful conservation of intact carnivore guilds. Such payments could be particularly valuable if they incentivized the formation of fully integrated conservancies.

### 5. Conclusions

Efforts to promote land uses that reduce costs associated with having carnivores, and confer financial value to those species are likely effective means of conserving them. Conserving lions and wild dogs outside of protected areas will be most challenging and may be dependent on promoting the formation of large conservancies where they can be reintroduced, or by achieving increased tolerance among landowners close to source populations. The higher tolerance of large carnivores among younger farmers (in keeping with the findings of [24], [31]), coupled with the spread of wildlife-based land uses in southern Africa allows for cautious optimism regarding the conservation of large carnivores outside of protected areas in the region.
